# 

**Published:** 1896-05

**Authors:** 


### CONTENTS.

ORIGINAL COMMUNICATIONS—

PRACTICAL PATHOLOGY OF THE MUCOUS MEMBRANE OF THE NOSE, THROAT, AND EARS OF INFANTS. By Thomas F. Rumbold, M.D., St. Louis, Mo. .......... 145

THE AFTER-TREATMENT OF TRACHEOTOMY CASES FOR MEMBRANOUS CROUP, WITH AN OBSERVATION OF THREE SUCCESSFUL, OUT OF FOUR OPERATIONS. By R. M. Harbin, M.D., Rome, Ga. .......... 154

PERSONAL AND SURGICAL REMINISCENCES OF THE WAR. By J. McFadden Gaston, M.D., Atlanta, Ga. .......... 161

MODERN METHODS IN THE TREATMENT OF SKIN DISEASES. By Bernard Wolf, M.D., Atlanta, Ga. .......... 171

REMARKS ON ABDOMINAL PTOSIS, VISCERO-PTOSIS, ENTERO-PTOSIS, OR GLENARD'S DISEASE. By Hugh M. Taylor, M.D., Richmond, Va. .......... 177

SOCIETY REPORTS—

THE MEDICAL ASSOCIATION OF GEORGIA .......... 182

EDITORIAL—

THE AMERICAN MEDICAL ASSOCIATION .......... 195

THE ASSOCIATION AT AUGUSTA .......... 197

THE CASE OF KITSON VS. PLAYFAIR .......... 198

SELECTIONS AND ABSTRACTS—

MISCELLANEOUS .......... 200

MEDICAL ITEMS .......... 208

BOOK REVIEWS .......... 214

ADVERTISERS' INDEX TO ADVERTISEMENTS .......... xvi

MEDICAL ITEMS—CONTINUED .......... x, xi, xii, xiii, xiv, xv

